# Fully percutaneous emergency management of an acute myocardial infarction complicated by papillary muscle rupture treated with percutaneous revascularization and mitral transcatheter edge-to-edge repair: a case report

**DOI:** 10.1093/ehjcr/ytag407

**Published:** 2026-06-02

**Authors:** Mauro Pennacchi, Francesco De Felice, Amedeo Pergolini, Annachiara Pingitore, Domenico Gabrielli

**Affiliations:** Department of Cardio-Thoraco-Vascular Disease, Division of Cardiology, San Camillo Hospital, Circ.ne Gianicolense 87, Rome 00152, Italy; Department of Cardio-Thoraco-Vascular Disease, Division of Cardiology, San Camillo Hospital, Circ.ne Gianicolense 87, Rome 00152, Italy; Department of Cardio-Thoraco-Vascular Disease, Division of Cardiology, San Camillo Hospital, Circ.ne Gianicolense 87, Rome 00152, Italy; Department of Cardio-Thoraco-Vascular Disease, Division of Cardiology, San Camillo Hospital, Circ.ne Gianicolense 87, Rome 00152, Italy; Department of Cardio-Thoraco-Vascular Disease, Division of Cardiology, San Camillo Hospital, Circ.ne Gianicolense 87, Rome 00152, Italy

**Keywords:** Papillary muscle, Acute myocardial infarction, Angioplasty, Mitraclip, Emergency, Echocardiography, Case report

## Abstract

**Background:**

Papillary muscle rupture (PMR) in a rare and life-threatening complication of a myocardial infarction (MI). In our case we describe a complete percutaneous emergency management of this complication with coronary angioplasty followed to successful MitraClip repair.

**Case summary:**

We report a case of a 62-year-old man with MI and severe mitral regurgitation. We performed percutaneous revascularization and intra-aortic balloon pump (IABP) was placed. After a transthoracic and transoesophageal echo evaluation, a PMR was diagnosed. Due to intractable pulmonary oedema, we proceed to emergency mitral edge-to-edge repair with two MitraClip just 2 h later. Mitral regurgitation was reduced to mild, and patient was extubated.

**Conclusion:**

This case reports a completely percutaneous management of an MI complicated by PMR in prohibitive surgical risk patient. Emergency treatment of MI and PMR with coronary angioplasty and MitraClip is feasible and could be a bail-out option in high surgical risk patients.

Learning points

- In the setting of acute myocardial infarction complicated by severe mitral regurgitation, a careful TEE evaluation of anatomical feasibility for transcatheter repair should be considered.
- Emergency treatment of papillary muscle rupture with transcatheter repair, if anatomically feasible, could be a bail-out option in high surgical risk patients.

## Introduction

A 62-year-old man was transferred to our hospital (A. O. San Camillo—Forlanini, Rome) from a spoke referral centre for non ST-elevation myocardial infarction (MI) complicated by resuscitated cardiac arrest and pulmonary oedema. The patient reported episodes of intermittent chest pain in the 2 days prior to admission to the emergency department. Prior medical history was silent; patient did not report any previous cardiovascular event. Patient’s cardiovascular risk factors were arterial hypertension and dyslipidaemia. The differential diagnoses could be acute ischaemic left ventricular dysfunction, ischaemic mitral regurgitation, ventricular septal defect, pericardial effusion/tamponade, and acute myocarditis.

## Summary figure

**Figure:**
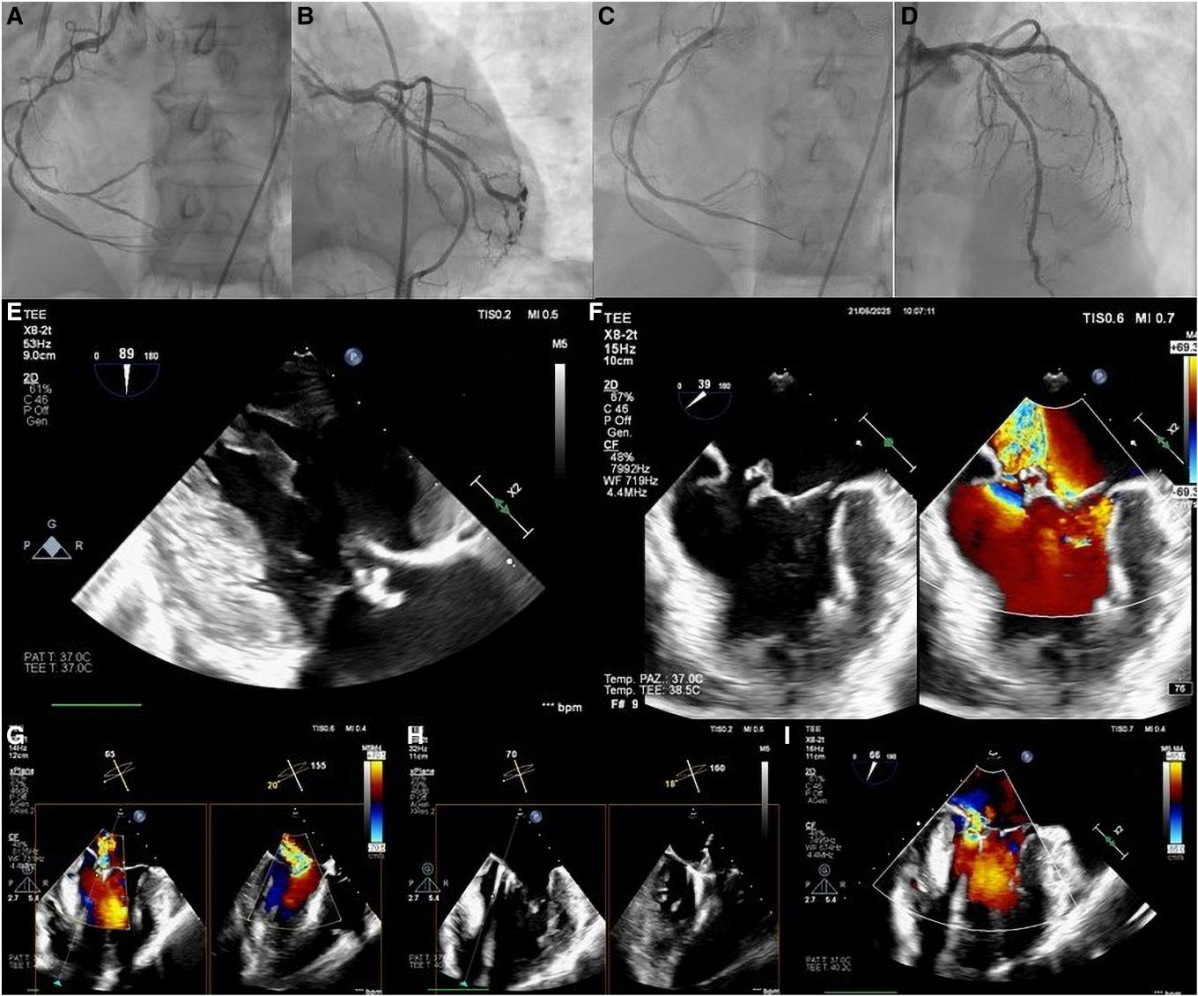
(A) Right coronary angiography with diffuse disease and subocclusion in distal tract. (B) Left coronary angiography with subocclusion of obtuse marginal branch and severe stenosis of proximal left anterior descending artery. (C) Right coronary post-angioplasty results and (D) left coronary post-angioplasty result. (E) TOE in *trans*-gastric view showing a mass attached to the prolapsing leaflet corresponding to postero-medial PM head detachment. (F) TOE bicommissural view showing flail leaflet, posterior leaflet tethering, and massive mitral regurgitation at colour Doppler. (G) A more than moderate residual mitral regurgitation from the antero-medial portion of the valve after the first mitraclip (XTW) implantation. (H) Second mitraclip (NTW) implanted medially to the first one, at the para-commissural A3-P3 scallop level. (I) Final result showing mild/mild-moderate mitral regurgitation, originating between the two clips. TOE, transoesophageal echo; PM, papillary muscle.

## Case presentation

At arrival, the patient was intubated, and at clinical examination, jugular venous distension and bilateral inspiratory crackles on lung auscultation were observed. There was a three out of six systolic apical murmur, without evident gallop rhythm and no peripheral oedema. In the spoke hospital, the patient underwent an incomplete bedside echocardiogram because of sudden cardiac arrest.

**Figure 1:**
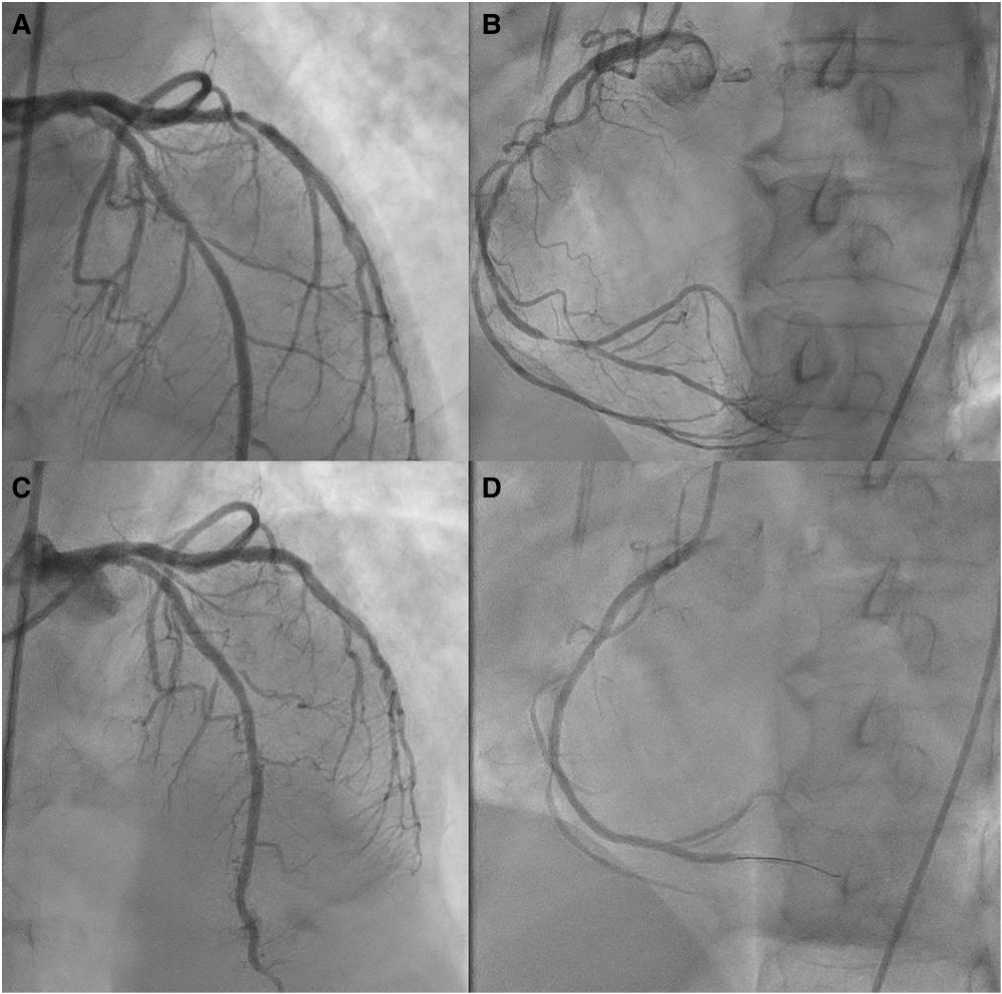
Basal and post-PCI coronary angiography. (*A*) RCA angiogram showing diffuse disease with multiple subocclusive stenosis. (*B*) LCA angiogram showing critical LAD stenosis and critical OMB stenoses. (*C*) RCA post-PCI. (*D*) LCA post-PCI in LAD and OMB. PCI, percutaneous coronary intervention, RCA, right coronary artery, LCA, left coronary artery, LAD, left anterior descending, OMB, obtuse marginal branch.

**Figure 2:**
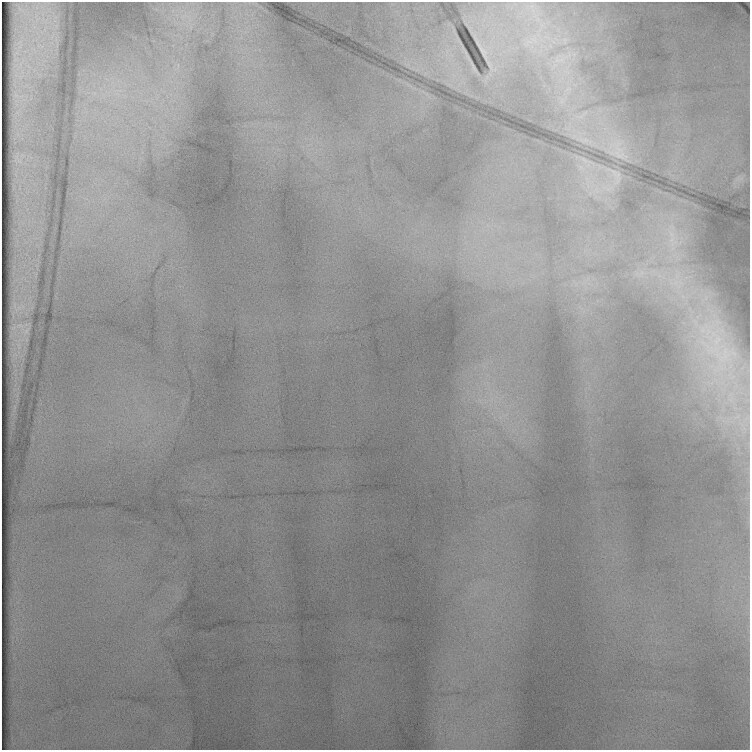
Intra- aortic balloon pump insertion.

**Figure 3:**
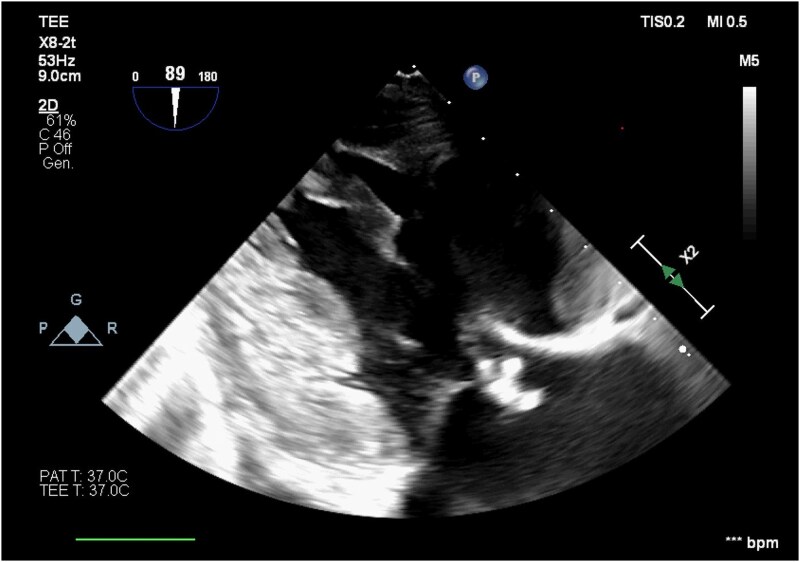
TOE in *trans*-gastric view showing a mass attached to the prolapsing leaflet corresponding to postero-medial PM head detachment. TOE, transoesophageal echo, PM, papillary muscle.

**Figure 4:**
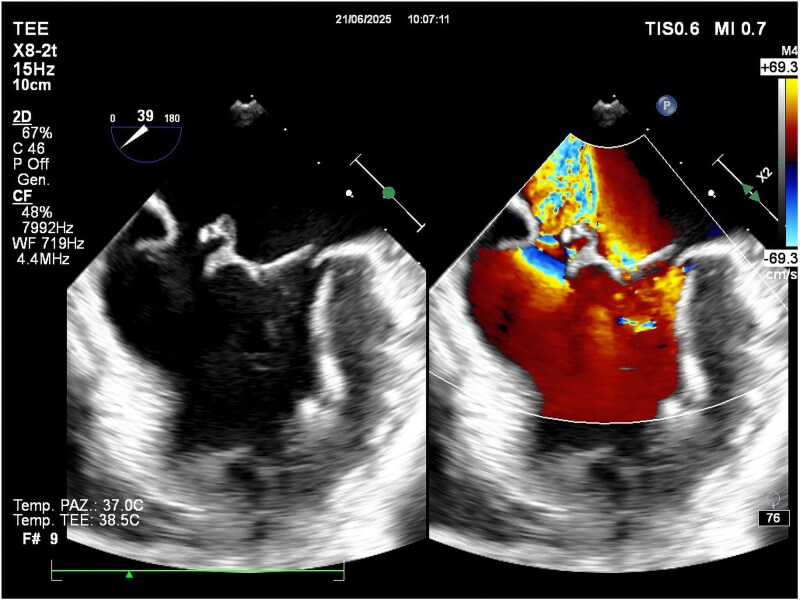
TOE bicommissural view showing flail leaflet, posterior leaflet tethering and massive mitral regurgitation at colour Doppler. TOE, transoesophageal echo.

Coronary angiography was performed first. Proximal left anterior descending artery, right coronary artery, and left circumflex with obtuse marginal branch were diffusely deceased and suboccluded. We did not recognize a clear culprit lesion, so a complete revascularization with drug-eluting stent implantation was successfully achieved (*Figure 1*) according to the European Society of Cardiology (ESC) Guidelines for the management of acute coronary syndromes with a IIa class of recommendation.^1^ Despite endotracheal intubation and intensive medical therapy, the pulmonary oedema was difficult to treat, requiring a fast echocardiogram in the cath-lab that showed severe mitral regurgitation and consequently, intra-aortic balloon pump (IABP) was placed (*Figure 2*). Patient was transferred to the intensive care unit where the transoesophageal echocardiography (TEE) documented massive mitral regurgitation, mainly due to anterior mitral leaflet (AML) flail involving the A2-A3 scallops, notably with a mass attached to the prolapsing leaflet corresponding to the near-complete detachment of the postero-medial papillary muscle (PM) head. Additionally, the tethering of posterior mitral leaflet, caused by ischaemic remodelling of the mid-basal infero-septal wall, further, worsened the mitral insufficiency. (see Supplementary material online, *Videos S1*–S4) (*Figures 3* and *4*). Although the anatomy was complex, the feasibility of transcatheter edge-to-edge repair (TEER) was supported by detailed echocardiographic assessment. Both the anterior and the posterior leaflet tissue resulted in sufficient length and coaptation reserve for stable clip grasping; moreover, the posterior leaflet mobility and thickness remained suitable for secure capture, despite its ischaemic tethering. Additionally, the postero-medial PM showed a small preserved accessory head maintaining partial chordal support, which ensured adequate leaflet stability after device implantation. Finally, the regurgitant jet was well localized and accessible from a transseptal approach.

The patient was promptly evaluated by the heart team and was considered at prohibitive surgical risk (STS score 34,1%). Considering the TEE feasibility for a M-TEER, although anatomy was challenging, the patient was scheduled for an emergent transcatheter repair, according to the actual ESC guidelines not yet published at the time of the procedure.^2^ Indeed, he was in intractable pulmonary oedema despite intubation; progressive desaturation occurred requiring elevated supplemental oxygen (100% FiO_2_) to maintain a saturation of 92%–93%, consistent with acute cardiogenic pulmonary oedema.

This is a clinical context of society for cardiovascular angiography and interventions stage C evolving cardiogenic shock, under norepinephrine support at 0.2 µg/kg/min. After IABP placement, norepinephrine was discontinued and dobutamine was initiated at 5 µg/kg/min.

An emergent transcatheter mitral valve edge-to-edge repair (MitraClip™ G4—Abbott) procedure was performed under the TE guidance. The first clip (XTW) was positioned at the A2M-P2M scallops level, in order to target the previously described wide regurgitation jet (see Supplementary material online, *Video S5*). Following device deployment, a more than moderate residual mitral regurgitation was observed, originating from the antero-medial portion of the valve (see Supplementary material online, *Video S6*). Therefore, a second clip (NTW) was implanted medially to the first one, at the para-commissural A3-P3 scallop level, obtaining a single orifice valve with transvalvular mean gradient of 3 mmHg^3^ (see Supplementary material online, *Video S7*). The final result demonstrated mild/mild-moderate mitral regurgitation, originating between the two clips (see Supplementary material online, *Video S8*). At the end of procedure, IABP was removed.

Within a few hours, the inotropic support was withdrawn and approximatively 24 h after hospital admission, the patient was extubated. During hospital stay, the patient started angiotensin-converting enzyme inhibitor, β-blockers, and statin other than double antiplatelet therapy with acetylsalicylic acid 100 mg and ticagrelor 90 mg bid planned for 12 months. Pre-discharge echocardiogram showed a mild-reduced ejection fraction and a mild-to-moderate mitral regurgitation. After 7 days, the patient was discharged. Three-month clinical follow-up was uneventful with the same medical therapy. The patient will have the next follow-up in 6 months.

## Discussion

Postinfarction PMR typically occurs within 5 days after MI and carries a mortality rate of 80% during the first week without emergency surgical interventions.^4^ Although surgery is the standard of care, more than half of patients are excluded because of prohibitive operative risk, and among those who undergo surgery, the operative mortality rate is as high as 40%.^5,6^

The postero-medial PM is affected more often than the antero-lateral PM because its blood flow is provided by the right coronary artery while the antero-lateral PM blood flow is supplied by both the diagonal branch of the left anterior descending artery and the left circumflex artery. This double blood supply explains why the antero-lateral PM is affected only in large anterior infarcts and the relative lower incidence of rupture compared to the posteromedial PM.^7^

Several cases described a transcatheter edge-to-edge repair for PMR with good haemodynamic results and clinical outcomes.

To achieve a good result is important, therefore, we need to carefully evaluate if the valve morphology is suitable for edge-to-edge repair and a heart valve team is available in a short time.

In our case, there was a completely percutaneous management of the coronary and structural disease in an emergency setting. This was something we could call as ‘primary percutaneous mitral repair’ similarly to primary angioplasty.

What we want to emphasize is the timing of mitral repair because in our opinion, there could be a positive correlation between a fast mitral repair with a faster haemodynamic recovery avoiding the complications that often arise if cardiogenic shock continues for hours or days such as renal impairment, multiorgan failure, or infection disease.

In our case, there was an intractable pulmonary oedema despite endotracheal intubation and intensive medical therapy that was sliding towards cardiogenic shock. We promptly performed the mitral repair, and as a result, IABP was removed at the end of procedure. The patient was extubated some hours later without inotropes administration.

## Conclusion

Papillary muscle rupture is a rare but life-threatening complication of MI. Transcatheter edge-to-edge repair can be a safe option in high surgical risk patients especially in the setting of a cardiogenic shock. A fast percutaneous repair may avoid the complications that occur with a prolonged cardiogenic shock, positively impacting the prognosis.

## Supplementary Material

ytag407_Supplementary_Data

## Data Availability

The data underlying this article are not publicly available due to patient privacy concerns but can be shared on reasonable request to the corresponding author.

## Lead author biography

Mauro Pennacchi completed his medical school training and obtained PhD at the Sapienza University of Rome. Currently, he is an interventional cardiologist working at the Cardio-Thoraco-Vascular Department of Azienda Ospedaliera San Camillo—Forlanini, Rome, Italy. His main field of interest is coronary and structural intervention.

## References

[1] Byrne RA, Rossello X, Coughlan JJ, Barbato E, Berry C, Chieffo A, et al 2023 ESC guidelines for the management of acute coronary syndromes: developed by the task force on the management of acute coronary syndromes of the European Society of Cardiology (ESC). Eur Heart J 2023;44:3720–3826. 37622654 10.1093/eurheartj/ehad191

[2] Praz F, Borger MA, Lanz J, Marin-Cuartas M, Abreu A, Adamo M, et al 2025 ESC/EACTS guidelines for the management of valvular heart disease: developed by the task force for the management of valvular heart disease of the European Society of Cardiology (ESC) and the European Association for Cardio-Thoracic Surgery (EACTS). Eur Heart J 2025;46:4635–4736.40878295

[3] De Felice F, Paolucci L, Musto C, Cifarelli A, Coletta S, Pennacchi M, et al Postprocedural trans-mitral gradient in patients with degenerative mitral regurgitation undergoing mitral valve transcatheter edge-to-edge repair. Catheter Cardiovasc Interv 2023;102:310–317. 37232290 10.1002/ccd.30698

[4] Wolff R, Cohen G, Peterson C, Wong S, Hockman E, Lo J, et al MitraClip for papillary muscle rupture in patient with cardiogenic shock. Can J Cardiol 2014;30:1461.e13–1461.e14. 10.1016/j.cjca.2014.07.015 25442448

[5] Bilge M, Alemdar R, Yasar AS. Successful percutaneous mitral valve repair with the MitraClip system of acute mitral regurgitation due to papillary muscle rupture as complication of acute myocardial infarction. Catheter Cardiovasc Interv 2014;83:E137–E140. 23592592 10.1002/ccd.24960

[6] Tyler J, Narbutas R, Oakley L, Ebinger J, Nakamura M. Percutaneous mitral valve repair with MitraClip XTR for acute mitral regurgitation due to papillary muscle rupture. J Cardiol Cases 2020;22:246–248. 33133320 10.1016/j.jccase.2020.07.001 PMC7588477

[7] Doi T, Nagura S, Fukahara K, Yoshimura N. Surgical treatment of complete anterolateral papillary muscle rupture following acute myocardial infarction. Ann Thorac Cardiovasc Surg 2014;20:926–928. 24429692 10.5761/atcs.cr.13-00171

